# A-Kinase Anchor Protein 1 deficiency causes mitochondrial dysfunction in mouse model of hyperoxia induced acute lung injury

**DOI:** 10.3389/fphar.2022.980723

**Published:** 2022-10-03

**Authors:** Ramani Soundararajan, Helena Hernández-Cuervo, Timothy M Stearns, Anthony J Griswold, Sahebgowda Sidramagowda Patil, Jutaro Fukumoto, Venkata Ramireddy Narala, Lakshmi Galam, Richard Lockey, Narasaiah Kolliputi

**Affiliations:** ^1^ Division of Allergy and Immunology, Department of Internal Medicine, Morsani College of Medicine, University of South Florida, Tampa, FL, United States; ^2^ University of South Florida, Department of Molecular Medicine, Morsani College of Medicine, Tampa, FL, United States; ^3^ Jackson Laboratory, Bar Harbor, ME, United States; ^4^ John P. Hussman Institute for Human Genomics, University of Miami, Miller School of Medicine, Miami, FL, United States; ^5^ Department of Zoology, Yogi Vemana University, Kadapa, AP, India

**Keywords:** Akap1, hyperoxia, mitochondrial dysfunction, Akt, Drp1, ETS

## Abstract

**Background:** Critically ill patients on supplemental oxygen therapy eventually develop acute lung injury (ALI). Reactive oxygen species (ROS) produced during ALI perturbs the mitochondrial dynamics resulting in cellular damage. Genetic deletion of the mitochondrial A-kinase anchoring protein 1 (Akap1) in mice resulted in mitochondrial damage, Endoplasmic reticulum (ER) stress, increased expression of mitophagy proteins and pro-inflammatory cytokines, exacerbating hyperoxia-induced Acute Lung Injury (HALI).

**Objective:** Despite a strong causal link between mitochondrial dysfunction and HALI, the mechanisms governing the disease progression at the transcriptome level is unknown.

**Methods:** In this study, RNA sequencing (RNA-seq) analysis was carried out using the lungs of *Akap1* knockout (*Akap1* ^−/−^) mice exposed to normoxia or 48 h of hyperoxia followed by quantitative real time PCR and Ingenuity pathway analysis (IPA). Western blot analysis assessed mitochondrial dysfunction, OXPHOS complex (I-V), apoptosis and antioxidant proteins. Mitochondrial enzymatic assays was used to measure the aconitase, fumarase, citrate synthase activities in isolated mitochondria from *Akap1* ^−/−^ vs. Wt mice exposed to hyperoxia.

**Results:** Transcriptome analysis of *Akap1* ^−/−^ exposed to hyperoxia reveals increases in transcripts encoding electron transport chain (ETC) and tricarboxylic acid cycle (TCA) proteins. Ingenuity pathway analysis (IPA) shows enrichment of mitochondrial dysfunction and oxidative phosphorylation in *Akap1* ^−/−^ mice. Loss of AKAP1, coupled with oxidant injury, significantly decreases the activities of TCA enzymes. Mechanistically, a significant loss of dynamin-related protein 1 (Drp1) phosphorylation at the protein kinase A (PKA) site Serine 637 (Ser637), decreases in Akt phosphorylation at Serine 437 (Ser47) and increase in the expression of pro-apoptotic protein Bax indicate mitochondrial dysfunction. Heme oxygenase-1 (HO-1) levels significantly increased in CD68 positive alveolar macrophages in *Akap1* ^−/−^ lungs, suggesting a strong antioxidant response to hyperoxia.

**Conclusion:** Overall these results suggest that AKAP1 overexpression and modulation of Drp1 phosphorylation at Ser637 is an important therapeutic strategy for acute lung injury.

## Introduction

Acute respiratory distress syndrome (ARDS) is a life-threatening condition and a common cause of respiratory failure in critically ill patients. They present clinically with bilateral infiltrates on chest x-ray, pulmonary edema non-cardiogenic in origin, and severe hypoxemia (Ashbaugh et al., 1967). The molecular mechanisms underlying ARDS pathogenesis remain unknown. The mortality rate of 30–40% is partially due to the lack of effective therapeutic strategies (Matthay et al., 2019). ARDS, according to the Berlin definition, can be categorized as mild, moderate, or severe based on the degree of hypoxemia (Ferguson et al., 2012). Acute lung injury (ALI) is the mild form of ARDS.

ALI in mice manifests as a diffused alveolar injury, characterized by pulmonary inflammation and edema (Ferguson et al., 2012). *In vivo* exposure of mice to ≥95% oxygen causes hyperoxia-induced acute lung injury (HALI), a model to study ALI (Kallet and Matthay, 2013). Reactive oxygen species (ROS) generated during hyperoxia induces oxidative stress and cause an increase in pro-inflammatory cytokines, immune cell infiltrates, and diffused alveolar edema and damage (Kolliputi and Waxman, 2009; Waxman and Kolliputi, 2009; Kolliputi et al., 2010). Excessive production of ROS lead to mitochondrial damage, including disruption of Ca^+2^ homeostasis, loss of mitochondrial membrane permeability and damage to the mitochondrial respiratory chain (Tait and Green, 2012).

Mitochondria, the “cell powerhouses,” are critical for ATP synthesis, serve as scaffolds to initiate protein-protein signaling, and regulate Ca^+2^ homeostasis and ROS (Roca-Portoles and Tait, 2021). Mitochondria also play an active role in autophagy, mitophagy, cell death signaling, and innate immunity (Pellegrino et al., 2014). AKAP1 is a Protein Kinase A (PKA) anchoring mitochondrial protein, with a N-terminal outer mitochondrial membrane (OMM) domain with a mitochondrial targeting sequence, PKA binding site, and c-terminal RNA binding KH and Tudor domains (Chen et al., 1997). AKAP1 has dual-specificity, binding to both RI and RII regulatory subunits of PKA (Huang et al., 1997). It is a scaffolding protein that forms a signaling hub by interacting with proteins and mRNAs at the OMM (Livigni et al., 2006). It also promotes cell survival by regulating mitochondrial dynamics (Liu et al., 2020).

The dynamic nature of mitochondria can be defined by the opposing events of mitochondrial fission and fusion (Mishra and Chan, 2014). The cytoplasmic dynamin-related GTPase Drp1 and fission protein Fis1 are key proteins that are involved in mitochondrial fission, while mitofusin 1 and 2 (Mfn1 and Mfn2) and optic atrophy 1 (Opa1) proteins play an important role in mitochondrial fusion (Lee and Yoon, 2016). The cAMP dependent PKA phosphorylates Drp1 at Serine 637 (Ser637) in an AKAP1 dependent manner to initiate mitochondrial fusion, whereas dephosphorylation by Ca^2+^-dependent phosphatase calcineurin (CaN) promotes mitochondrial fission in neuronal cells (Dickey and Strack, 2011; Merrill et al., 2011). For example, loss of AKAP1 causes a reduction in Drp1 phosphorylation at Ser637 and is associated with glaucomatous retinal ganglion cell (RGC) dysfunction (Edwards et al., 2020). The role of AKAP1-mediated phosphorylation or dephosphorylation at Ser637 in HALI is unknown.

*Akap1* ^*−/−*^ mice are a valuable tool to dissect the role of AKAP1 in cardiovascular, neurodegenerative diseases and cancer (Liu et al., 2020). In 2018, our group demonstrated that *Akap1* ^*−/−*^ mice exposed to 48 h of hyperoxia display ALI, characterized by increases in pro-inflammatory cytokines, edema, inflammatory immune cells, aberrant mitochondria, and autophagy and mitophagy (Narala et al., 2018). Further, *Akap1* ^*−/−*^ mice subjected to oxidant injury exhibit higher expression of ER stress markers including binding immunoglobulin protein 1 (Bip1), C/EBP Homologous protein (CHOP), eIF2α phosphorylation, JNK phosphorylation and ER stress-induced autophagy (Sidramagowda Patil et al., 2022).

The signal transduction pathways modulated by AKAP1 to promote cell survival in lungs exposed to high oxygen levels is unknown. RNA-sequencing analysis of the transcriptome changes in the lungs of *Akap1* ^*−/−*^ mice exposed to hyperoxia led to evaluate the effect of AKAP1 loss on mitochondrial dynamics and function.

## Materials and methods

### Study approval

The study was performed in accordance with the regulations set forth by the University of South Florida Institutional Animal Care and Use Committee, IACUC (Animal Welfare Assurance Number: A4100-01) and in the “Guide for the Care and Use of Experimental Animals” established by the National Institute of Health (NIH) (1996, Revised 2011).

### Mouse studies

C57BL/6 mice were purchased from (Envigo Indianapolis, IN, United States). *Akap1* ^*−/−*^ mice (C57BL/6 background) were a gift from Dr. Stefan Strack (University of Iowa, United States). *Akap1* ^*−/−*^ mice were bred with C57BL/6 mice (Envigo Indianapolis, IN, United States) to produce *Akap1* ^+/−^ heterozygous mice. The heterozygous male and female mice were bred to generate *Akap1* ^−/−^ and Wt littermate controls that were used for all experiments. Both male and female mice, 7–9 weeks of age were used in the study. Mice were maintained in a specific pathogen-free environment under a 12 h dark-light cycle and fed a standard diet *ad libitum*.

### Hyperoxia exposure

*Akap1* ^*−/−*^ (*n* = 3–6) and *Wt* (*n* = 3–6) mice were exposed to room air (normoxia, 21% oxygen) or >95% oxygen (48 h hyperoxia) in cages in an airtight chamber (75 × 50 × 50 cm, Biospherix Parish, NY, United States) that was fitted with proOx p100 sensor (Sidramagowda Patil et al., 2020). Mice were euthanized using intraperitoneal injection of Ketamine/Xylazine (100 mg/ml), followed by cervical dislocation. Lungs were perfused with ice-cold PBS.

### RNA sequencing analysis

Total RNA was extracted from *Wt* and *Akap1* ^*−/−*^ lungs (normoxia and hyperoxia groups) using RNeasy Mini Kit^®^ (Qiagen, Germantown, Maryland, United States) as described (Soundararajan et al., 2015). The total RNA quality was assessed using the Agilent 2100 Bioanalyzer. RNA-seq was performed at the John P. Hussman Institute for Human Genomics, Center for Genome Technology Sequencing Core as described (Soundararajan et al., 2015). Briefly, 600 ng of total RNA was used as input for the KAPA RNA HyperPrep with RiboErase HMR kit per the manufacturer’s instructions to create ribosomal RNA-depleted sequencing libraries. Each sample was sequenced to more than 30 million raw paired end 100 bp reads on the Illumina NovaSeq 6000. Sequencing data was processed through a bioinformatics pipeline including quality control, alignment to the mm10 mouse reference genome with STAR aligner v2.5.2a and gene quantification performed with the Gene Counts STAR function against the GENCODE version 19 annotation gene dataset. Resulting count data was input into edgeR software for differential expression analysis. Counts were normalized using the trimmed mean of M-values (TMM) method to account for sequencing amount difference between the libraries (Robinson and Oshlack, 2010). Differential expression was performed within edgeR using generalized linear models with the quasi-likelihood F-test that provides strict error rate control by accounting for the uncertainty in gene dispersion estimation. Genes with a false discovery rate *p*-value (FDR) ≤ 0.05 and the average log counts per million across all the samples ≥0 were considered differentially expressed.

### Ingenuity pathway analysis

DEGs were analyzed by Ingenuity pathway analysis (IPA)’s Core Analysis pipeline with default settings (Ingenuity Systems, 2021 Fall release, Qiagen). For identifying pathways enriched in *Akap1* ^*−/−*^ hyperoxia group relative to *Wt* hperoxia group, we overlaid the DEGs in *Akap1* ^*−/−*^ normoxia versus *Akap1* ^*−/−*^ hyperoxia dataset with DEGs from *Wt* Normoxia versus *Wt* hyperoxia dataset. In this way, the OXPHOS and TCA networks and Tables were generated for *Akap1* ^*−/−*^ mice and *Wt* mice exposed to hyperoxia.

### qRT-PCR

Total RNA from mouse samples used for RNA-sequencing as well as from independent experiments were used for Quantitative Real Time PCR (qRT-PCR) analysis to increase the rigor. 1 μg of total RNA was reverse transcribed using the IScript cDNA synthesis kit (Bio-Rad, Hercules, California, United States) as described (Soundararajan et al., 2016). qRT-PCR was performed using cDNA and Taqman probes; Mm01207872_m1 (Ndufb9), Mm00481172_m1 (Sdhc), Mm00445961_m1 (Uqcrc2), Mm07298317_g1 (Cox7b), Mm00503351_m1 (Ndufa8) and Mm00431960_m1 (Atp5a1) using TaqMan Fast Advanced Master Mix in Quant Studio 3 PCR System (Thermo Fisher) according to the manufacturer’s instructions (Thermofisher Scientific, Carlsbad, CA, United States). As an internal control, we used the Taqman probe Mm02619580_g1 for Actb. A relative fold change (RFC) in transcripts was calculated using the Biorad CFX Manager software using comparative CT method (Delta CT) and expressed as 2^−ddCT^.

### Western blot analyses

Whole lung lysate was prepared from *Wt* and *Akap1* ^*−/−*^ lungs as described (Soundararajan et al., 2016; Fukumoto et al., 2019). The primary antibodies and the dilutions used for the western blot analyses are listed in Table 1. The antibodies used in this study were validated by vendors and supported in the literature. Briefly, 10 μg of total protein was separated on SDS-PAGE and electroblotted onto polyvinylidene difluoride (PVDF) membrane. The membranes were incubated with primary antibodies at 4°C overnight and dilutions done as described (Table 1). The membranes were probed with anti-mouse or anti-rabbit HRP-conjugated secondary antibody (1:4000, Jackson Immunoresearch Laboratory, West Grove, PA, United States) at room temperature for 1 h. The Kwik Qwant ECL system (Kindle Biosciences, Greenwich, CT) was used to detect the protein. HRP-conjugated β-Actin antibody (Sigma Aldrich, St Louis, MO) acted as a loading control. Images were acquired using the FUJIFILM camera (X-A2). Post-processing of the images was performed using Adobe Photoshop CS6. Quantitation of the protein band was performed using Image J software. Band intensity was normalized to β-Actin and the protein expression was expressed as a fold change.

**TABLE 1:** List of Primary Antibodies used in this study.

Primary antibodies	Dilutions	Source	Identifier
Akt, Rabbit polyclonal	1:1000	Cell Signaling technology	4691
p-Akt (S437), Rabbit polyclonal	1:1000	Cell Signaling technology	4060
Akap1, Rabbit polyclonal	1:1000	Cell Signaling technology	5203S
HO-1, rabbit polyclonal	1:1000	Cell Signaling technology	70081S
CD68, mouse monoclonal	1:1000	Santa Cruz	sc-20060
Drp1, Rabbit polyclonal	1:1000	Cell Signaling technology	8570
p-Drp1 (S637), Rabbit polyclonal	1:1000	Cell Signaling technology	4867S
SOD2, Rabbit polyclonal	1:1000	Cell Signaling technology	13141S
Rodent OXPHOS antibody cocktail	1:500	Abcam	ab110413
Mfn1, mouse monoclonal	1; 1000	Abcam	ab57602
Fis1, Rabbit polyclonal	1:1000	Thermo Fisher Scientific	PA522142
Opa1, Rabbit polyclonal	1:1000	Novus biologicals	NB110-55290
Bax, Rabbit polyclonal	1:1000	Cell Signaling technology	2772T
β-Actin-HRP, Rabbit	1:10,000	Cell Signaling technology	4970
β-Actin-HRP, Mouse	1:10,000	Cell Signaling technology	12262

### Histology and immunofluorescence

Lungs were perfused with ice cold PBS and the left lung was expanded and fixed using a 5 ml solution of 4% PFA for 48 h, followed by standard histological processing and stained with hematoxylin and eosin (Fukumoto et al., 2019). We used 5 µm paraffin-embedded lung sections for immunofluorescence (IF) analysis. Lung sections were incubated with the primary antibodies HO-1 (1:200), Bax (1:200), and CD68 (1:100) overnight at 4°C. For double IF, on day 1, lung sections were incubated with primary antibody (Rabbit HO-1, 1:100) overnight at 4°C. On day 2, lung sections were washed with 1X PBS three times for 5 min and incubated with Goat anti-rabbit Cy3 (1:200, Jackson Immunoresearch Laboratory) at room temperature for 1 h. After PBS washes, the lung sections were incubated with primary antibody (Mouse CD68, 1:100) overnight at 4°C. On day 3, the lungs sections were washed and incubated with anti-mouse 488 (1:200, Jackson Immunoresearch Laboratory) at room temperature for 1 h. After PBS washes, lung sections were counterstained using Vectashield containing DAPI (Vector Laboratories, Burlingame, CA). Image acquisition and processing: The fluorescent images were captured on the Olympus U-TV0.63XC, SN1E25675 microscope (Tokyo, Japan) and DP21 camera model U-LH100HG SN1G18573 utilizing the cellSens Dimension XV Image processing software. The images were saved as high resolution Tiff images (300 dpi) and processed using Adobe Photoshop version CS6. Quantitation of images: To quantify HO-1, CD68 and Bax staining, 3-5 independent, non-overlapping images (40x) were used per sample and various parameters were acquired using Fiji (Image J, NIH) software. The formula: CTCF = Integrated Density-(Area of the selected cell x Mean fluorescence of background readings) was used to calculate the corrected total cell fluorescence (CTCF) as described (McCloy et al., 2014).

### TUNEL staining

Wt and *Akap1* ^*−/−*^ paraffin-embeded lung sections (normoxia and hyperoxia groups) were subjected to TUNEL staining using One-step TUNEL *in situ* Apoptosis kit as per manufacturer’s instructions (E-CK-A320, Elabsciences, Houston, Tx). Briefly, following deparaffinization and hydration steps, lung sections were treated with Proteinase K for 20 min at 37°C. For positive control, lung section was treated with DNAase I and for negative control, lung section was treated with only DNAase buffer I at 37°C for 30 min. The sections were equilibrated with TdT equilibration buffer at 37°C for 30 min followed by staining with labelling solution (FITC and TdT enzyme in TdT equilibration buffer) at 37°C for 60 min in dark. Following PBS wash, sections were counterstained using Vectashield containing DAPI (Vector Laboratories, Burlingame, CA) and imaged as previously described.

### Mitochondrial enzymatic assays

Mitochondria were isolated from *Wt* and *Akap1* ^*−/−*^ lungs using the mitochondria isolation kit as per the manufacturer’s instructions (Thermo Scientific). Mitochondrial pellets were lysed in 2% CHAPS in TBS supplemented with protease and phosphatase inhibitors, quantified by Pierce BCA protein assay kit (Thermo Scientific) and stored at −80°C. The fumarase activity colorimetric assay kit MAK206 (Sigma-Aldrich, St. Louis, MO), citrate synthase activity kit MAK193 (Sigma-Aldrich) and aconitase activity assay kit MAK051 (Sigma-Aldrich) were used to detect enzymatic activity in *Wt* and *Akap1* ^*−/−*^ mitochondrial fraction (25 μg) according to the manufacturer’s instructions. Aconitase activity (nmole/min/ml), citrate synthase activity (milliunit/mL) and fumarase activity (milliunit/mL) was normalized to the amount of protein used in the assay. Mitochondrial Complex I activity was determined using 25 μg of mitochondrial lysate and Complex I enzyme activity kit (#ab109721, Abcam, Waltham, MA) as per manufacturer’s instructions. The Complex I activity (mOD/min) was normalized to the amount of protein used in the assay.

### Statistical analysis

Data were expressed as mean ± S.E.M (Standard error mean). GraphPad Prism version 10 and IBM SPSS Statistics (v26) was used for statistical analysis. For analysis of two groups, two-tailed, unpaired Student’s t-test was used. For analysis of more than 2 groups, one-way ANOVA and Tukey’ post-hoc test was used. To test the effect of two independent variables on a dependent variable, two-way ANOVA was used (IBM SPSS). A level of *p* < 0.05 was considered statistically significant.

## Results

### Phenotypic characterization of *Akap1* ^*−/−*^ mice

The *Akap1* ^−/−^ mice were generated by Newhall et al. (2006) by targeting central exon 2 as it encodes the majority of the protein in all splice variants (3 out of the 6 splice variants of *Akap1* gene encode functional protein). Genotyping of *Akap1* heterozygous (*Akap1* ^*+/−*^) mice revealed the presence of a *Wt* allele (600 bp) and a mutant allele (400 bp) (Supplementary Figure S1A). Breeding of *Akap1* ^*+/−*^ mice generated *Akap1* ^*−/−*^ and *Wt* littermate controls in the Mendelian ratio (Supplementary Figure S1A). The genotyping of mice (Wt and *Akap1* ^−/−^ normoxia and hyperoxia, *n* = 3 per group) used for RNA-seq analysis is shown in Supplementary Figure S1A. RNA-seq analysis revealed significant increase in *Akap1* transcript level in *Akap1* ^−/−^ mice exposed to hyperoxia versus its normoxia controls, respectively (Supplementary Figure S1B, *p < 0.05 versus *Akap1* ^−/−^ normoxia control). Western blot analysis revealed a significant increase in AKAP1 protein level in Wt hyperoxia versus Wt normoxia controls (Supplementary Figure S1C, ***p < 0.001 versus Wt nornmoxia). However, there was a significant decrease in AKAP1 protein expression in *Akap1* ^−/−^ hyperoxia relative to Wt hyperoxia validating previous finding (Narala et al., 2018) (Supplementary Figure S1C, ***p < 0.001 versus Wt hyperoxia). Histological examination of lungs derived from *Akap1* ^*−/−*^ mice exposed to 48 h of hyperoxia shows alveolar congestion and thickening, and infiltration of immune cells versus normoxia controls (Supplementary Figure S1D).

### Transcriptome analysis of *Akap1* gene reveals widespread mutations

RNA-sequencing was used to profile the transcriptome changes in *Akap1* ^*−/−*^ and *Wt* lungs under normoxia and hyperoxia. Visualization of sequence alignment *via* Integrated Genomics Viewer (IGV) of *Akap1* gene shows a significant decrease or absence of reads at exon 2 (Box) and frameshift mutations in other exons in *Akap1* ^*−/−*^ relative to *Wt* controls (Supplementary Figure S2). These data indicate that excision of exon 2 occurs only in *Akap1* ^−/−^ group and this causes a frameshift mutation in all other exons, whereas Wt controls show reads at exon 2 and no frameshift mutations in rest of the exons (Supplementary Figure S2). As exon 2 encodes majority of AKAP1 protein, deletion of this exon impacts the function of the protein.

### Identification of differentially expressed genes (DEG) in *Akap1* ^*−/−*^ mice exposed to hyperoxia

**FIGURE 1:**
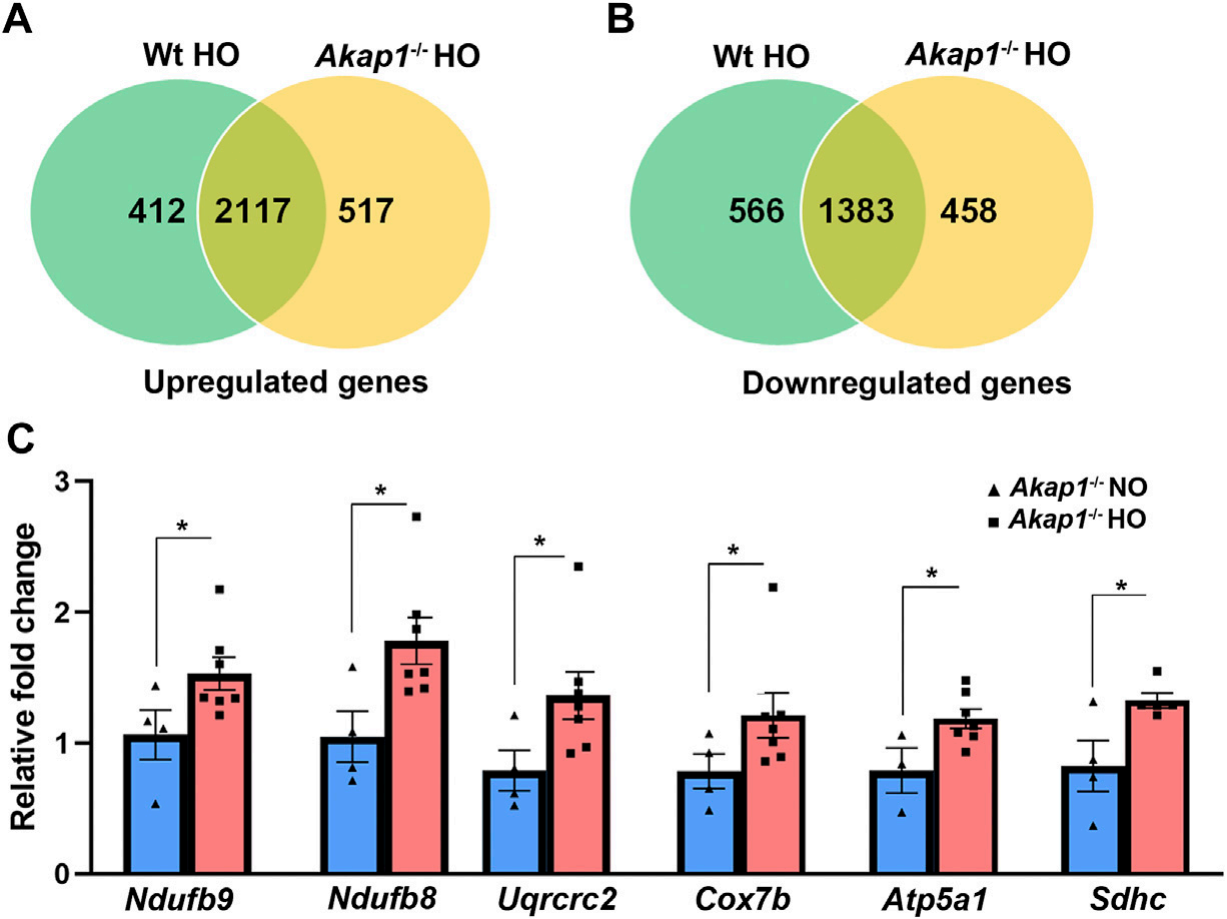
Differentially expressed genes in *Akap1* ^*−/−*^ and *Wt* mice exposed to hyperoxia. **(A)** Venn diagram depicts upregulated genes unique and commonly shared between the *Wt* hyperoxia group and *Akap1* ^*−/−*^ hyperoxia group and **(B)** Venn diagram depicts downregulated genes unique and commonly shared between the *Wt* hyperoxia group and *Akap1* ^*−/−*^ hyperoxia group, respectively. **(C)** qRT-PCR analysis of transcripts (ETC and TCA) in the *Akap1* ^*−/−*^ hyperoxia group relative to *Akap1* ^*−/−*^ normoxia group (*n* = 6 mice per group). Data represented as mean ± SEM. Two-tailed unpaired Student’s t-test. *p < 0.05 *Akap1* ^*−/−*^ hyperoxia versus *Akap1* ^*−/−*^ normoxia control.

Within *Akap1* ^*−/−*^ and *Wt* sample groups, RNA-sequencing analysis revealed 5,212 and 4,982 DEG’s, respectively, in the pairwise comparison of hyperoxia versus normoxia groups (Supplementary Tables S1, S2, respectively). A venn diagram shows 3,046 upregulated genes in hyperoxia; of these, 412 were upregulated in *Wt* hyperoxia group, 517 were upregulated in the *Akap1* ^*−/−*^ hyperoxia group, and 2,117 genes overlapped between the groups (Figure 1A). Similarly, RNA-seq analysis identified 2,407 downregulated genes in hyperoxia; of these 566 were downregulated in the *Wt* hyperoxia group, 458 were downregulated in the *Akap1* ^*−/−*^ hyperoxia group, and 1,383 overlapped between the groups (Figure 1B).

### Ingenuity Pathway Analysis reveals mitochondrial dysfunction in *Akap1* ^*−/−*^ mice exposed to hyperoxia

Comparison of DEGs from the *Akap1* ^*−/−*^ hyperoxia versus *Akap1* ^*−/−*^ normoxia dataset with DEGs from the *Wt* hyperoxia versus *Wt* normoxia dataset identified the top canonical pathways in both genotypes. The top canonical pathways that are significantly enriched in *Akap1* ^*−/−*^ mice exposed to hyperoxia include mitochondrial dysfunction, oxidative phosphorylation, the tricarboxylic citric acid (TCA) cycle, eIF2, mTOR, and sirtuin signaling (Table 2). The top canonical pathways significantly enriched in Wt mice exposed to hyperoxia include calcium-induced T lymphocyte apoptosis, Nur77 signaling in T lymphocytes, the Th1 and Th2 activation pathways, the Th2 pathway, and glycogen degradation II (Table 3).

**TABLE 2:** Ingenuity Canonical Pathways enriched in *Akap1* ^*−/−*^ mice exposed to hyperoxia.

Ingenuity canonical pathways	-log (*p* Value)	Molecules
Mitochondrial dysfunction	15.7	NDUFA4,COX6B1,NDUFB4,COX7B,NDUFA9,NDUFA7,Cox6c,ATP5F1A,XDH,ACO2,TRAK1,ATP5MG,SDHC, NDUFAF2,NDUFA2,UQCRB,Atp5e,PDHA1,ATP5F1C,NDUFA5,NDUFB9,COX11,NDUFV2,NDUFA11,NDUFA6,UQCRC2,NDUFS6,ATP5MF,SDHD, VDAC1,ATP5MC3,NDUFA8
Oxidative Phosphorylation	15.6	NDUFA4,NDUFB4,COX6B1,COX7B,NDUFA9,NDUFA7,Cox6c,ATP5F1A,ATP5MG,SDHC, NDUFA2,UQCRB,Atp5e,ATP5F1C,NDUFB9,NDUFA5,COX11,NDUFV2,NDUFA11,NDUFA6,UQCRC2,NDUFS6,ATP5MF,SDHD,ATP5MC3,NDUFA8
Sirtuin Signaling Pathway	6.92	NDUFA4,PPARA, HIST1H1C,NDUFA9,NDUFA7,NDUFA2,PDHA1,NDUFA5,NDUFB9,GABARAPL1,PRKAA2,NDUFS6,NAMPT,ATG16L2,NDUFA8,NDUFB4,ATP5F1A,TUBA4A,SDHC, NDUFAF2,ATP5F1C,Hist1h1e, NDUFV2,NDUFA11,NDUFA6,UQCRC2,SDHD, VDAC1
EIF2 Signaling	6.73	RAP1B,EIF2S3,EIF3E,RPS29,RPL9,EIF3M,RPL14,EIF3F,RPL8,RPL35,RPS20,RPL13,KL,RPS13,EIF4A3,PIK3R6,RPL19,RPS25,RPS5,RPS12,RPS3,RPS17,RPL31,EIF3L
Regulation of eIF4 and p70S6K Signaling	4.85	RAP1B,EIF2S3,EIF3E,RPS29,EIF3M,EIF3F,RPS20,RPS13,KL,EIF4A3,PIK3R6,RPS25,RPS5,RPS12,RPS3,RPS17,EIF3L
mTOR Signaling	4.08	RAP1B,PLD3,EIF3E,RPS29,EIF3M,EIF3F,RPS20,RPS13,KL,EIF4A3,PRKAA2,PIK3R6,RPS25,RPS5,RPS12,RPS3,RPS17,EIF3L
TCA Cycle II (Eukaryotic)	3.15	CS,DHTKD1,ACO2,SDHD, SDHC

**TABLE 3:** Ingenuity Canonical Pathways enriched in Wt mice exposed to hyperoxia.

Ingenuity canonical pathways	-log (*p* Value)	Molecules
Calcium-induced T Lymphocyte Apoptosis	5.12	CD3G,CD3E,HLA-DMA,CD4,HLA-DMB, NR4A1,ATP2A3,HLA-DQB1,CD3D
Nur77 Signaling in T Lymphocytes	4.59	CD3G,CD28,CD3E,HLA-DMA,HLA-DMB,NR4A1,HLA-DQB1,CD3D
Th1 and Th2 Activation Pathway	4.48	CD3E,KLRD1,CD4,IKZF1,CXCR3,IL12RB2,HLADQB1,CD3D,CD3G,CD28,HLA-DMA,HLA-DMB,ACVR1C,IL2RB
Th2 Pathway	4.15	CD28,CD3G,CD3E,HLADMA,CD4,IKZF1,HLA-DMB,IL12RB2, HLA DQB1,ACVR1C,CD3D,IL2RB
Glycogen Degradation II	3.97	PGM2L1,PGM5,PYGB, PYGL

AKAP1 is an important mitochondrial scaffold and signaling protein and its loss most likely modulates mitochondrial gene expression in response to oxidant injury. A large majority of genes encoding Complex I to V proteins in the ETC (OXPHOS) pathway increased significantly in the *Akap1* ^*−/−*^ hyperoxia group versus *Akap1* ^*−/−*^ normoxia controls (Supplementary Table S3). Most of the genes show a relative fold change less than 2.0 with the exception of *Ndufb4* (fold change of 6.8). The genes unique to the *Akap1* ^*−/−*^ hyperoxia group include *Atp5j*, *Ndufa3*, *Ndufb10*, *Ndufb4*, *Ndufs7*, *Ndufs8* and *Uqcr11* (Supplementary Table S3). In contrast, in *Wt* mice exposed to hyperoxia, no unique genes in the OXPHOS pathway were identified (Supplementary Table S3). Genes encoding proteins in the TCA cycle significantly increased in the *Akap1* ^*−/−*^ hyperoxia group (Table 2), whereas in Wt mice exposed to hyperoxia, the transcripts encoding proteins in the TCA cycle were not altered (Table 3). This suggests that Akap1 deletion and oxidant injury modulates transcripts in the electron transport chain (ETC) and TCA cycle. The comparison of Wt normoxia versus *Akap^1-/-^* normoxia groups is shown in Supplementary Table S4, whereas comparison of Wt hyperoxia versus *Akap^1-/-^* hyperoxia groups is shown in Supplementary Table S5.

### Gene transcripts in the ETC and TCA pathways significantly increased in the lungs of *Akap1* ^*−/−*^ mice exposed to hyperoxia

Some of the genes upregulated in the *Akap1* ^*−/−*^ hyperoxia group relative to the *Akap1* ^*−/−*^ normoxia group were verified by qRT-PCR (relative fold change 1.5–1.82) and include *Ndufb9* (complex I), *Ndufb8* (complex I); *Uqcrc2* (complex III); *Cox7b* (complex IV); *Atp5a1* (complex IV) and *Sdhc* (complex II) (Figure 1C).

### Loss of AKAP1 decreases TCA enzyme activities in hyperoxia

**FIGURE 2:**
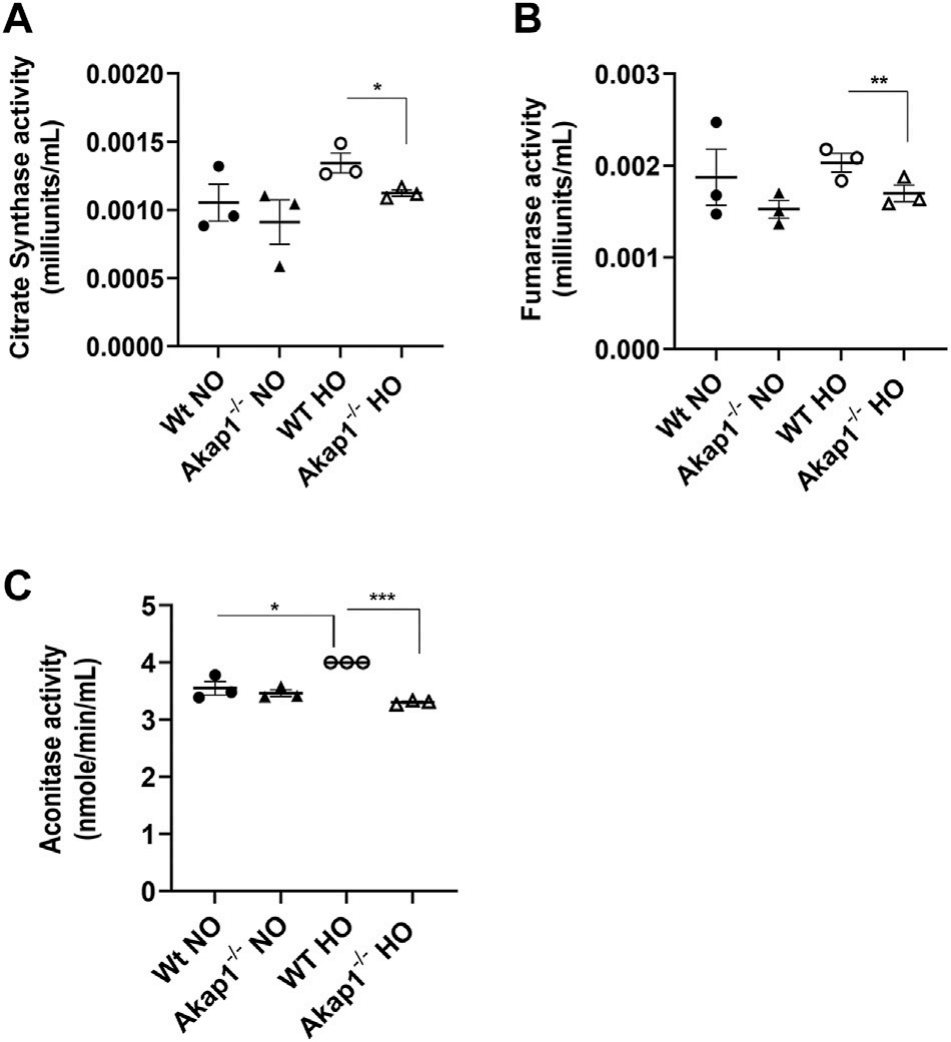
Decrease in mitochondrial enzymatic activities in *Akap1* ^*−/−*^ mice exposed to hyperoxia. **(A)** Citrate synthase activity (milliunits/mL) in the mitochondrial lysates from *Wt* and *Akap1* ^*−/−*^ mice exposed to normoxia or hyperoxia, respectively. **(B)** Fumarase activity (milliunits/mL) in mitochondrial lysates from *Wt* and *Akap1* ^*−/−*^ mice exposed to normoxia or hyperoxia, respectively. **(C)** Aconitase activity (milliunits/mL) in the mitochondrial lysates from *Wt* and *Akap1* ^*−/−*^ mice exposed to normoxia or hyperoxia. Data represented as mean ± SEM. Two-way ANOVA was used for analysis. *p < 0.05 *Akap1* ^*−/−*^ hyperoxia versus *Wt* hyperoxia control. **p < 0.01 *Akap1* ^−/−^ hyperoxia versus *Wt* hyperoxia control. ***p < 0.001 *Akap1* ^*−/−*^ hyperoxia versus *Wt* hyperoxia control. *p < 0.05 *Wt* hyperoxia versus *Wt* normoxia control.

Citrate synthase (CS), fumarase and aconitase activities were determined in the mitochondrial lysates to assess if AKAP1 loss affected the function of TCA enzymes in hyperoxic conditions. CS activity was not significantly upregulated in *Wt* hyperoxia group versus the *Wt* normoxia group (Figure 2A). Similarly, the CS activity was unchanged in the *Akap1* ^*−/−*^ normoxia versus *Akap1* ^*−/−*^ hyperoxia group. Under normoxia, CS activity between the *Wt* normoxia and *Akap1* ^*−/−*^ normoxia groups did not change. This suggests no contribution of genotype in normoxia. However, under hyperoxia, CS activity decreased significantly in the *Akap1* ^*−/−*^ versus *Wt* controls (Figure 2A, *p < 0.05 versus *Wt* controls) indicating an additive effect of genotype and oxidant injury. Similarly, fumarase activity significantly decreased in the *Akap1* ^*−/−*^ hyperoxia group relative to *Wt* hyperoxia controls (Figure 2B, **p < 0.01 versus *Wt* control) indicating an additive effect of genotype and oxidant injury. Fumarase activity did not change in the *Wt* group vs. the *Akap1* ^*−/−*^ group exposed to normoxia (Figure 2B). Similarly, fumarase activity did not change in the *Wt* normoxia versus *Wt* hyperoxia group and the *Akap1* ^*−/−*^ normoxia versus *Akap1* ^*−/−*^ hyperoxia group (Figure 2B). The results obtained for aconitase activity showed a difference when compared to CS or fumarase activity for the two groups. The aconitase activity was unchanged in the *Wt* normoxia group relative to the *Akap1* ^*−/−*^ normoxia group (Figure 2C). The mitochondrial aconitase activity was significantly lower in *Akap1* ^*−/−*^ mice exposed to hyperoxia versus *Wt* hyperoxia control (Figure 2C, ***p < 0.001 versus *Wt* control). There was a significantly higher aconitase activity in the *Wt* hyperoxia versus the *Wt* normoxia group (Figure 2C, *p < 0.05 versus *Wt* normoxia control) suggesting that this may be a compensatory mechanism to combat the effect of ROS on energy metabolism. However, the aconitase activity was unchanged in the *Akap1* ^*−/−*^ normoxia relative to the *Akap1* ^*−/−*^ hyperoxia group (Figure 2C). This indicates that a combination of Akap1 deletion and oxidative stress contributes to a significant decrease in aconitase activity. Collectively, the data suggest that the deletion of mitochondrial *Akap1* has a deleterious effect on the enzymes of the TCA cycle under hyperoxic conditions.

### *Akap1* deletion exacerbates oxidative stress in hyperoxia

**FIGURE 3:**
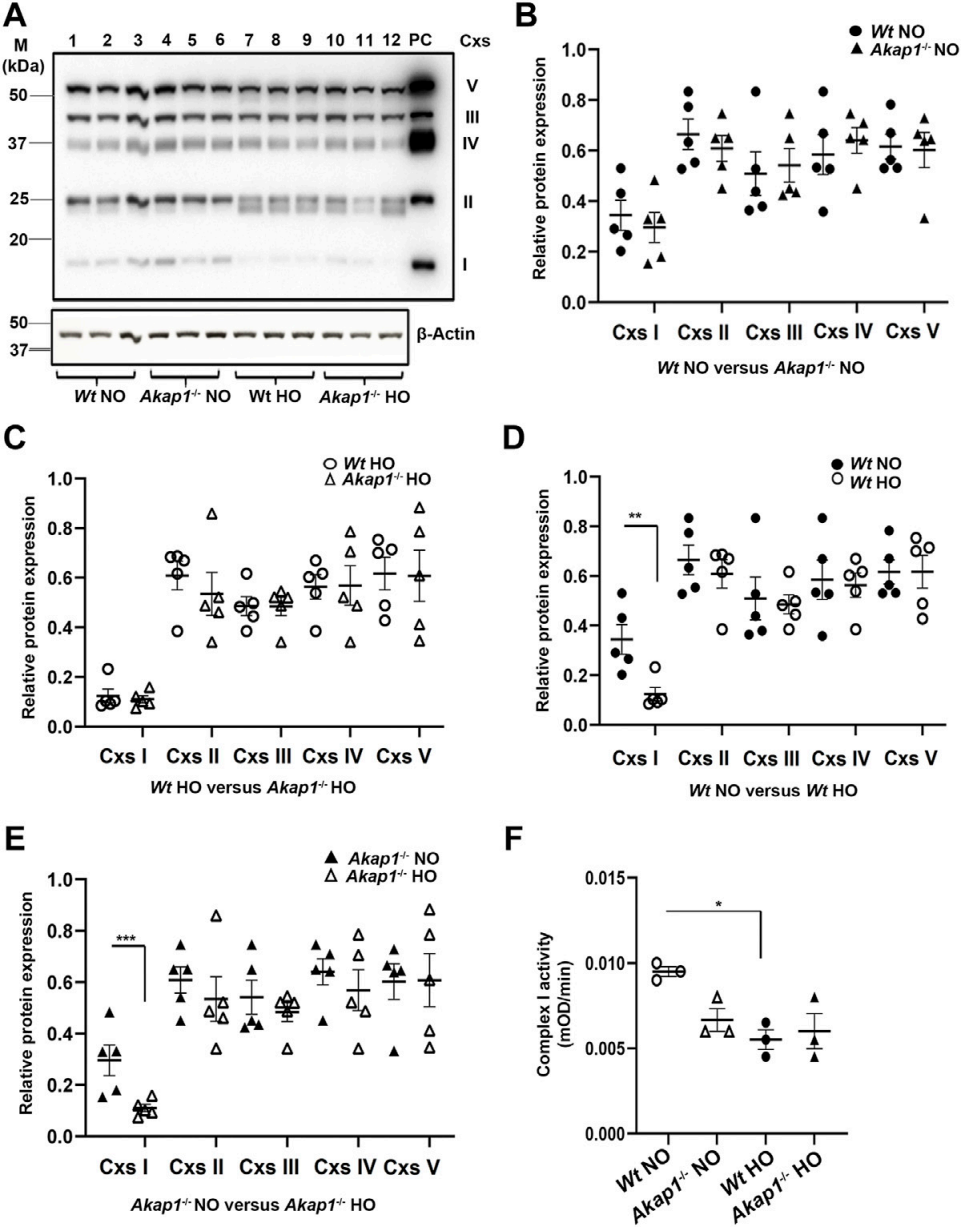
AKAP1 loss causes OXPHOS and Complex I dysfunction in hyperoxia. **(A)** Western blot analyses for OXPHOS Complexes in the lung lysates of *Wt* and *Akap1* ^*−/−*^ exposed to normoxia or hyperoxia. A representative image is shown. PC: Positive control (Rat heart mitochondrial lysate). Data normalized to β-Actin (loading control). **(B)** Quantitation of Complexes I to V in *Wt* normoxia vs. *Akap1* ^*−/−*^ normoxia. **(C)** Quantitation of Complexes I to V in *Wt* hyperoxia vs. *Akap1* ^*−/−*^ hyperoxia. **(D)** Quantitation of Complexes I to V in *Wt* normoxia vs. *Wt* hyperoxia. **p < 0.01 versus *W*t hyperoxia controls **(E)** Quantitation of Complexes I to V in *Akap1* ^*−/−*^ normoxia vs. *Akap1* ^*−/−*^ hyperoxia. ***p < 0.001 versus *Akap1* ^*−/−*^ hyperoxia. Relative protein expression of Complexes 1-V was calculated after normalization to β-Actin. **(B–E)** Data represented as mean ± SEM. *n* = 5 mice per group. Two-tailed unpaired Student’s t-test. Closed circle (*Wt* normoxia), closed triangle (*Akap1* ^*−/−*^ normoxia), open circle (*Wt* hyperoxia) and open triangle (*Akap1* ^*−/−*^ hyperoxia). **(F)** Complex I activity was assayed in the mitochondrial lysates from *Akap1* ^*−/−*^ normoxia and *Akap1* ^*−/−*^ hyperoxia groups. Complex I activity (mOD/min) was calculated. *n* = 3 mice per group. *p < 0.01 *Wt* hyperoxia versus Wt normoxia. One way ANOVA and post-hoc Tukey test was used for analysis. NO = normoxia. HO = hyperoxia.

OXPHOS complex (I-V) expression was determined in the whole lung lysates from *Akap1* ^*−/−*^ and *Wt* mice exposed to normoxia or hyperoxia to determine if *Akap1* loss exacerbates hyperoxia-induced mitochondrial dysfunction (Narala et al., 2018). Complex I expression changed in the *Wt* and *Akap1* ^*−/−*^ groups in normoxia relative to hyperoxia (Figures 3A,D,E). There was a significant decrease in complex I expression in *Wt* hyperoxia versus *Wt* normoxia controls (Figures 3A,D, **p < 0.01). Similarly, Complex I expression decreased significantly in the *Akap1* ^*−/−*^ hyperoxia group relative to *Akap1* ^*−/−*^ normoxia controls (Figures 3A,E, ***p < 0.001). This indicates that Complex I expression is affected by oxygen concentration irrespective of the genotype. Expression of Complexes II, III, IV and V were unchanged in the *Wt* and *Akap1* ^*−/−*^ groups in normoxia or hyperoxia (Figures 3A–E). Next, Complex I activity was determined in the mitochondrial lysates from *Wt* and *Akap1* ^*−/−*^ mice exposed to hyperoxia and normoxia. Hyperoxia results in a significant lower Complex I activity in *Wt* mice (Figure 3F, *p < 0.05). In contrast, Complex I activity between hyperoxia and normoxia exposed *Akap1* ^*−/−*^ mice was not affected indicating that *Akap1* deletion does not dysregulate Complex I activity under hyperoxia (Figure 3F).

**FIGURE 4:**
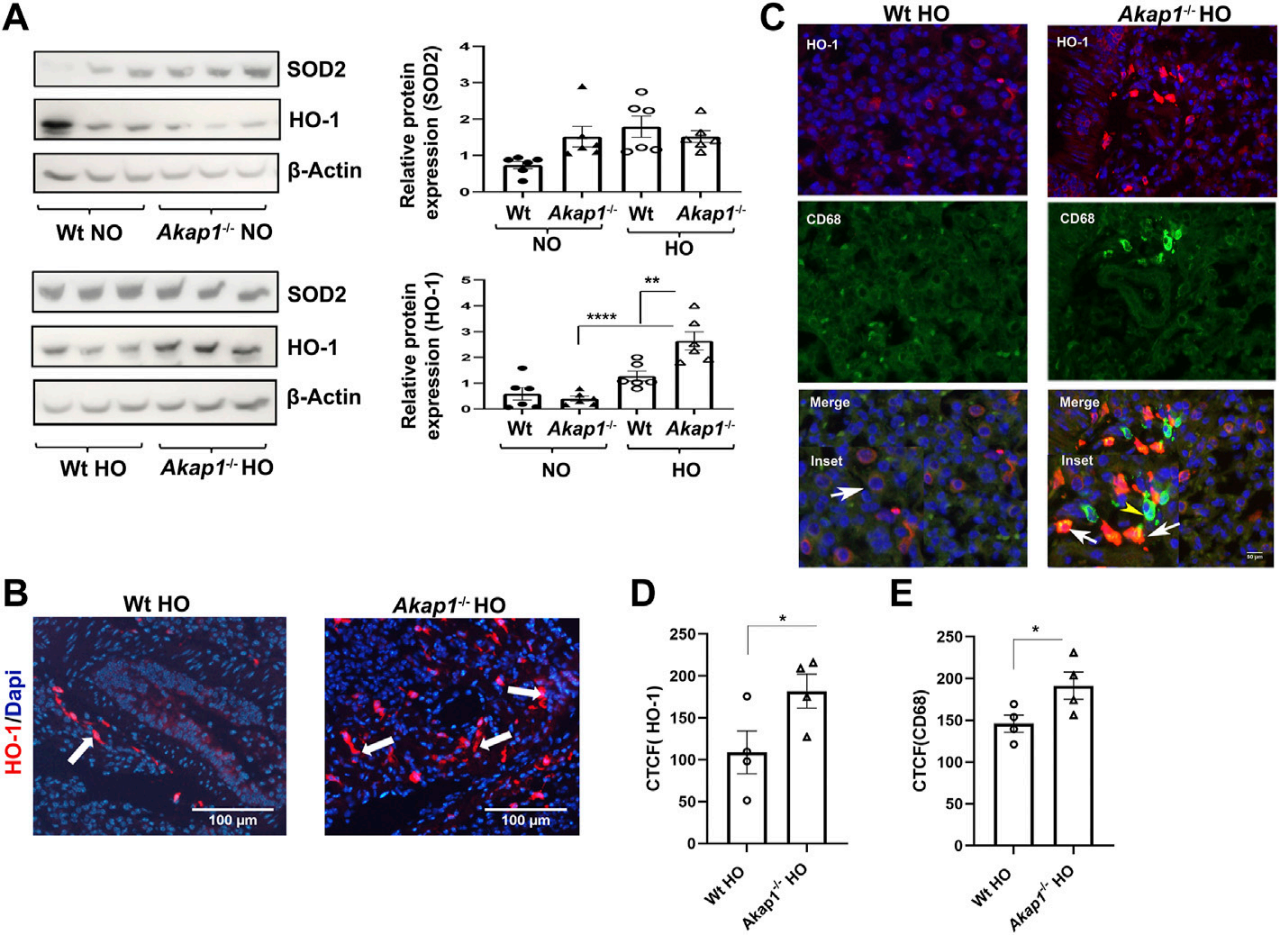
Oxidative stress increases in *Akap1* ^*−/−*^ mice exposed to hyperoxia. **(A)** Western blot analyses of SOD2 and HO-1 expression in the lysates from *Wt* and *Akap1* ^*−/−*^ mice exposed to normoxia or hyperoxia. SOD2 and HO-1 expression were quantified after normalization with β-Actin (loading control). **p < 0.01 *Akap1* ^*−/−*^ hyperoxia versus *Wt* hyperoxia. ****p < 0.0001 *Akap1* ^*−/−*^ hyperoxia versus *Akap1* ^*−/−*^ normoxia. *n* = 6 mice per group. Two-tailed unpaired Student’s t-test. **(B)** Representative images from IF analysis shows increased expression of HO-1 (white arrows) in the lung section of *Akap1* ^*−/−*^ hyperoxia versus *Wt* hyperoxia mice. Magnification: ×400. Scale bar: 100 μm. **(C)** Representative images from IF analyses of HO-1 (red) and CD68 (green) in lung sections derived from *Wt* and *Akap1* ^*−/−*^ mice exposed to hyperoxia. Arrowheads indicates the HO-1 positive cells co-labelled with CD68 (Macrophage marker). Arrow shows HO-1 expressing cells without CD68 expression. Nuclei stained with DAPI (blue). Magnification: ×400, scale bar: 50 μm. **(D)** Quantitative analysis of HO-1 expression is represented as corrected total cell fluorescence (CTCF). HO-1 expression in the lung sections of *Akap1* ^*−/−*^ hyperoxia versus *Wt* hyperoxia. *n* = 4 mice per group. Data represented as mean ± SEM. *p < 0.05 versus *Wt* hyperoxia. Unpaired Student’s t-test. *n* = 4 per group. **(E)** Quantitative analysis of CD68 ^+^ expression is represented as CTCF. CD68 ^+^ staining in the lung sections of *Akap1* ^*−/−*^ hyperoxia versus *Wt* hyperoxia. n = 4 mice per group. Data represented as mean ± SEM. *p < 0.05 *Akap1* ^*−/−*^ hyperoxia versus *Wt* hyperoxia. Unpaired Student’s t-test.

Hyperoxia induced ROS causes oxidative stress in mice (Kallet and Matthay, 2013). In order to determine if *Akap1* ^*−/−*^ mice exposed to hyperoxia activate the endogenous anti-oxidant pathway, the protein expression of the oxidative stress markers, mitochondrial manganese superoxide dismutase 2 (SOD2) and Heme oxygenase 1 (HO-1) were evaluated. SOD2 expression was unchanged between the *Wt* normoxia and *Wt* hyperoxia groups (Figure 4A). Similarly, there was no difference in SOD2 expression in the *Akap1* ^*−/−*^ normoxia versus *Akap1* ^*−/−*^ hyperoxia groups (Figure 4A). This indicates that the SOD2 pathway is unaffected by genotype or oxygen concentration.

HO-1 expression was determined in *Wt* and *Akap1* ^*−/−*^ mice exposed to normoxia and hyperoxia. There is a significantly higher HO-1 expression in the *Akap1* ^*−/−*^ mice exposed to hyperoxia versus *Akap1* ^*−/−*^ normoxia group (Figure 4A, ****p < 0.0001). In addition, HO-1 expression is significantly higher in the *Akap1* ^*−/−*^ hyperoxia compared to *Wt* hyperoxia groups (Figure 4A, **p < 0.01). This suggests an additive effect of *Akap1* deletion and oxygen concentration contributing to an increase in HO-1 expression in *Akap1* ^*−/−*^ mice. IF analysis of lung sections from the *Akap1* ^*−/−*^ hyperoxia group shows significant increase in the numbers of HO-1 positive cells in the alveolar spaces relative to *Wt* hyperoxia controls (Figures 4B,D, *p < 0.05). Double IF analysis reveals that the majority of CD68 ^+^ alveolar macrophages exhibit HO-1 positive staining (Figure 4C). Quantitation of IF images show a significant increase in CD68 ^+^ cells in the *Akap1* ^*−/−*^ hyperoxia versus *Wt* hyperoxia groups (Figure 4D, *p < 0.05). The conclusion from these results is that the loss of AKAP1 in response to hyperoxia activates the anti-oxidant pathway.

### *Akap1* deletion decreases phosphorylation of Drp1 at Ser637 under oxidative stress

**FIGURE 5:**
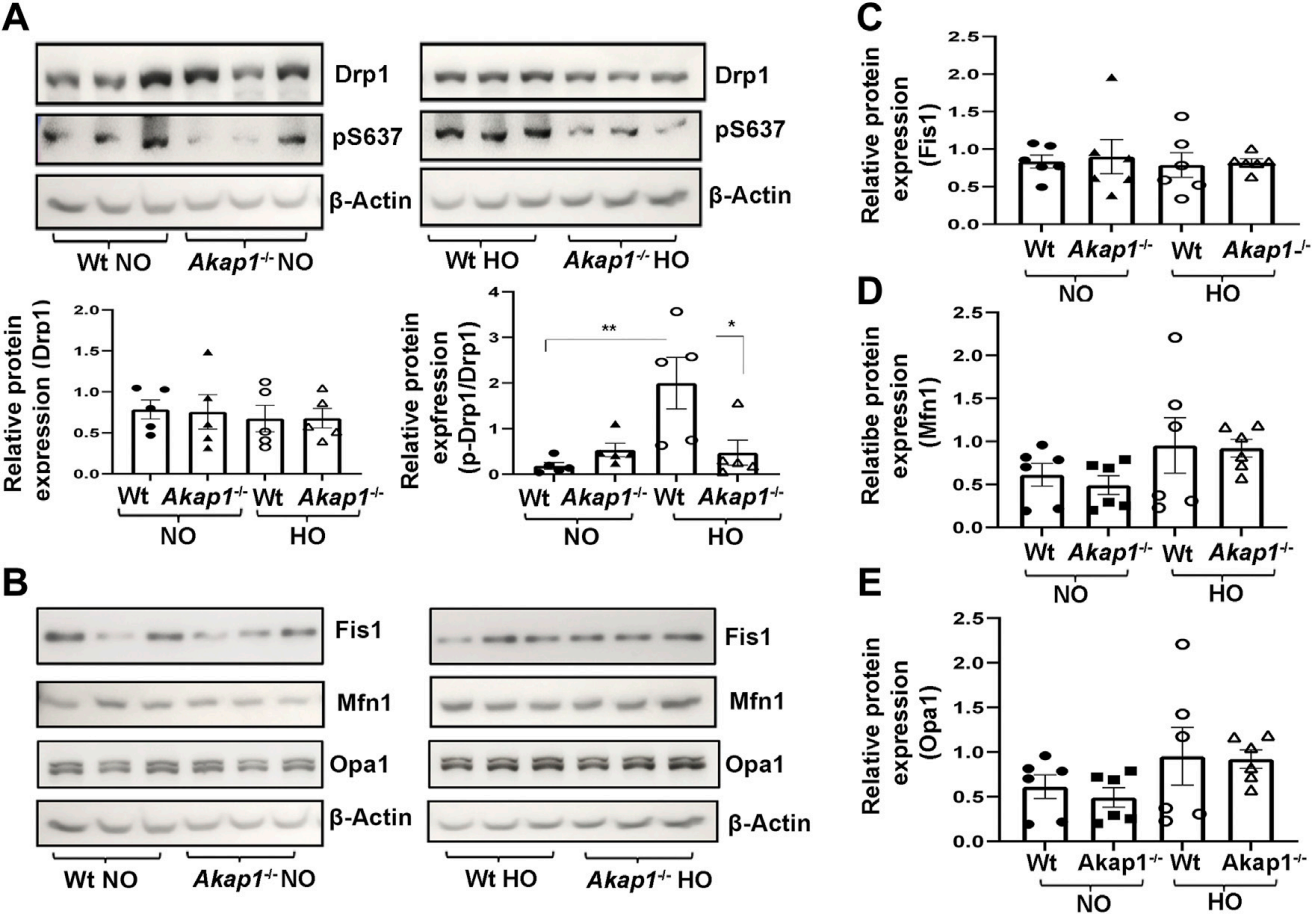
*Akap1* deletion decreases the phosphorylation of Drp1 at Ser637 under oxidative stress without affecting other fission or fusion protein expression. **(A)** Representative Western blot of total Drp1 and phosphorylated Drp1 (pS637) expression in the lung lysates derived from *Wt* and *Akap1* ^*−/−*^ mice exposed to normoxia or hyperoxia. Total Drp1 and pDrp1/Total Drp1 expression were quantitated after normalization with β-Actin control. Data represented as mean ± SEM. *n* = 5 mice per group. One-way ANOVA and post-hoc Tukey test. *p < 0.05 *Wt* hyperoxia versus *Wt* normoxia. **p < 0.01 *Akap1* ^−/−^ hyperoxia versus *Wt* hyperoxia control. **(B)** Representative Western blots of Fis1, Mfn1 and Opa1 expression in the lung lysates derived from *Wt* and *Akap1* ^*−/−*^ mice exposed to normoxia or hyperoxia. **(C–E)** Quantitative expression of Fis1, Mfn1 and Opa1 after normalization with β-Actin control. Data represented as mean ± S.E.M. *n* = 6 mice per group. NO = normoxia. HO = hyperoxia.

**FIGURE 6:**
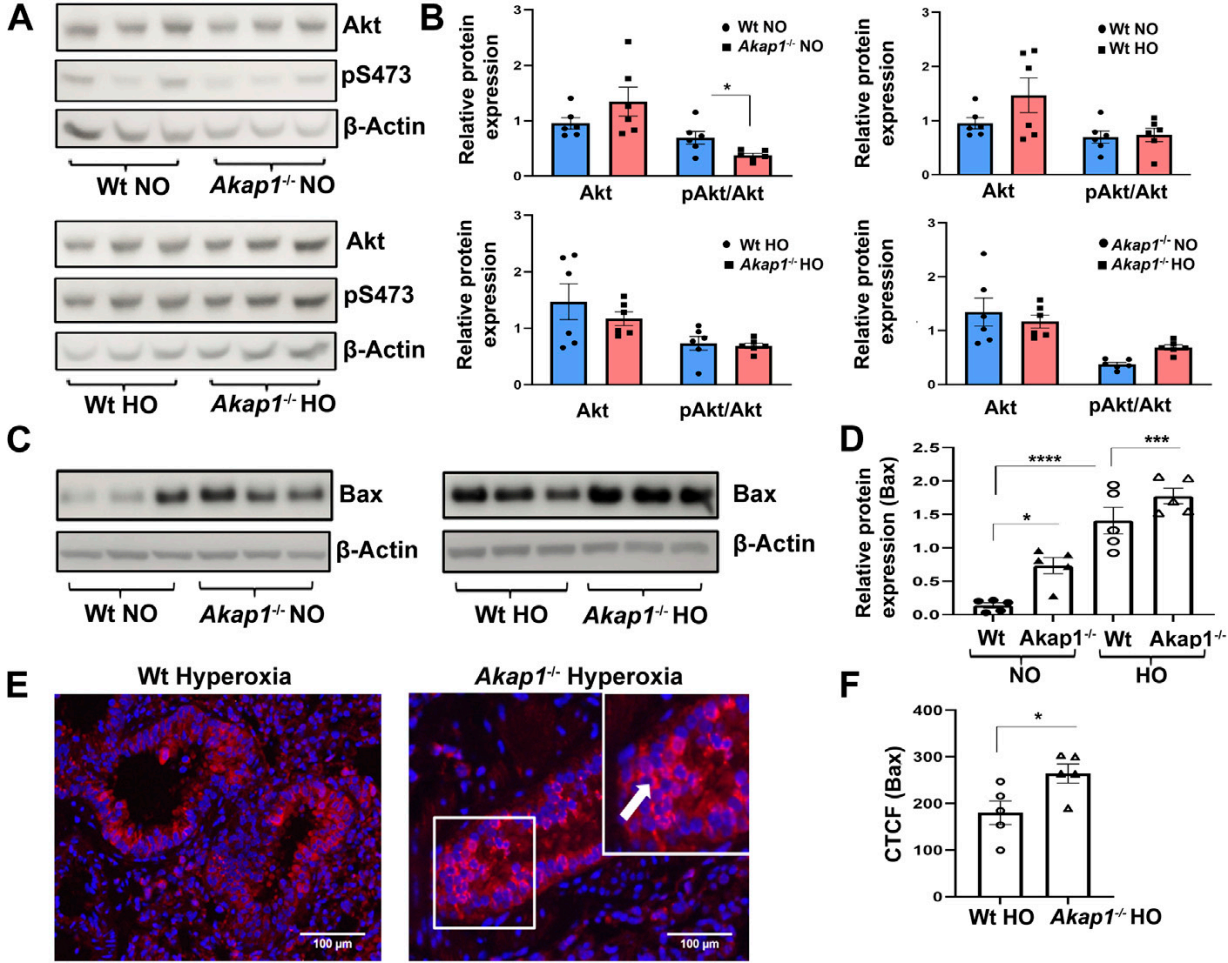
AKAP1 loss induced Akt inactivation and increased Bax expression under hyperoxia. **(A)** Representative Western blot analysis of total Akt and phosphorylated Akt (pS473) in the lung lysates derived from *Wt* and *Akap1* ^*−/−*^ mice exposed to normoxia or hyperoxia. **(B)** Total Akt and pAkt/Total Akt quantitated after normalization with β-Actin control. Data represented as mean ± SEM. *n* = 5 mice per group. Two-tailed unpaired Student’s t-test. *p < 0.05 *Akap1* ^−/−^ normoxia to *Wt* normoxia. ***p < 0.001 *Akap1* ^*−/−*^ normoxia versus *Akap1* ^*−/−*^ hyperoxia. **(C)** Representative Western blot analysis of Bax in the lung lysates from *Wt* and *Akap1* ^*−/−*^ mice exposed to normoxia or hyperoxia. **(D)** Quantitation of Bax expression after normalization with β-Actin control. Data presented as mean ± S.E.M. *n* = 5 mice per group. Two-tailed unpaired Student’s t-test. *p < 0.05 *Akap1* ^−/−^ normoxia versus *Wt* normoxia, ****p < 0.0001 Wt normoxia versus Wt hyperoxia and ***p < 0.001 *Akap1* ^*−/−*^ hyperoxia versus *Akap1* ^*−/−*^ normoxia. **(E)** Representative IF image of Bax expression in the lung section of *Wt* and *Akap1* ^*−/−*^ mice exposed to hyperoxia. White arrow shows positive Bax immunoreactivity. Nuclei stained with DAPI (blue). Magnification: ×400, scale bar: 50 μm. **(F)** Quantitative analysis of Bax expression is represented as CTCF. Bax immunoreactivity in the lung sections of *Akap1* ^*−/−*^ hyperoxia versus *Wt* hyperoxia. Data represented as mean ± SEM. *n* = 5 mice per group. Unpaired two-tailed Student’s t-test. *p < 0.05 *Akap1* ^−/−^ hyperoxia versus *Wt* hyperoxia. NO = normoxia. HO = hyperoxia.

Total Drp1 expression did not change in *Wt* or *Akap1* ^*−/−*^ mice exposed to hyperoxia versus normoxia controls (Figure 6A). Next, the levels of the phosphorylated form of Drp1 (Ser637) was studied. There was a significant upregulation of phospho-Drp1 (Ser637) expression in the *Wt* hyperoxia group versus *Wt* normoxia controls (Figure 5A, **p < 0.01). The expression of phospho-Drp1 (Ser637) failed to increase in *Akap1* ^*−/−*^ mice post-hyperoxia in comparison to *Wt* hyperoxia controls (Figure 5A, *p < 0.05), whereas it was unchanged in normoxia (Figure 5A). Fis1 protein expression was unchanged in the *Wt* or *Akap1* ^*−/−*^ groups in normoxia or hyperoxia (Figures 5B,C). Similarly, the expression of mitochondrial fusion protein, Mfn1, was unchanged in *Wt* and *Akap1* ^*−/−*^ hyperoxia lungs relative to their respective normoxia controls (Figures 5B,D). Opa1 expression did not change in *W*t or *Akap1* ^*−/−*^ lungs exposed to hyperoxia or normoxia (Figures 5B,E).

### AKAP1 loss inactivates Akt and activates Bax induced apoptosis in hyperoxia

The expression of Akt protein and its phosphorylation at Ser473 was determined in the lung lysate of *Wt* and *Akap1* ^*−/−*^ mice exposed to normoxia or hyperoxia. Total Akt expression did not change in *Wt* or *Akap1* ^*−/−*^ lungs in normoxia or hyperoxia (Figures 6A,B). pAkt (Ser473) levels did not change in the *Wt* hyperoxia group versus normoxia controls (Figures 6A,B). There is a significant lower level of pAkt protein in *Akap1* ^*−/−*^ lungs versus *Wt* lungs in normoxia (Figures 6A,B, *p < 0.05). Furthermore, there is a significant higher level of pAkt protein in the *Akap1* ^*−/−*^ hyperoxia versus the *Akap1* ^*−/−*^ normoxia group (Figures 6A,B, ***p < 0.001). Because inactivation of Akt triggers the intrinisic apoptotic pathway, the effect of AKAP1 loss on pro-apoptotic protein, Bax was determined in hyperoxia and normoxia. Bax expression increased significantly in normoxia upon AKAP1 loss relative to *Wt* controls (Figures 6C,D,*p < 0.05). There was a significant increase in Bax expression in the lungs of *Wt* and *Akap1* ^*−/−*^ mice exposed to hyperoxia versus their respective normoxia controls (Figures 6C,D, ****p < 0.0001, ***p < 0.001, respectively). Further, Bax expression was higher in the *Akap1* ^*−/−*^ hyperoxia group relative to *Wt* hyperoxia controls (Figures 6C,D). More specifically, bronchial epithelial cells and alveolar cells showed a significant increase in Bax immunostaining in *Akap1* ^*−/−*^ hyperoxia lung sections versus *Wt* hyperoxia controls (Figures 6E,F, *p < 0.05). These results indicate that AKAP1 loss and hyperoxia exposure induce Bax-mediated apoptosis.

**FIGURE 7:**
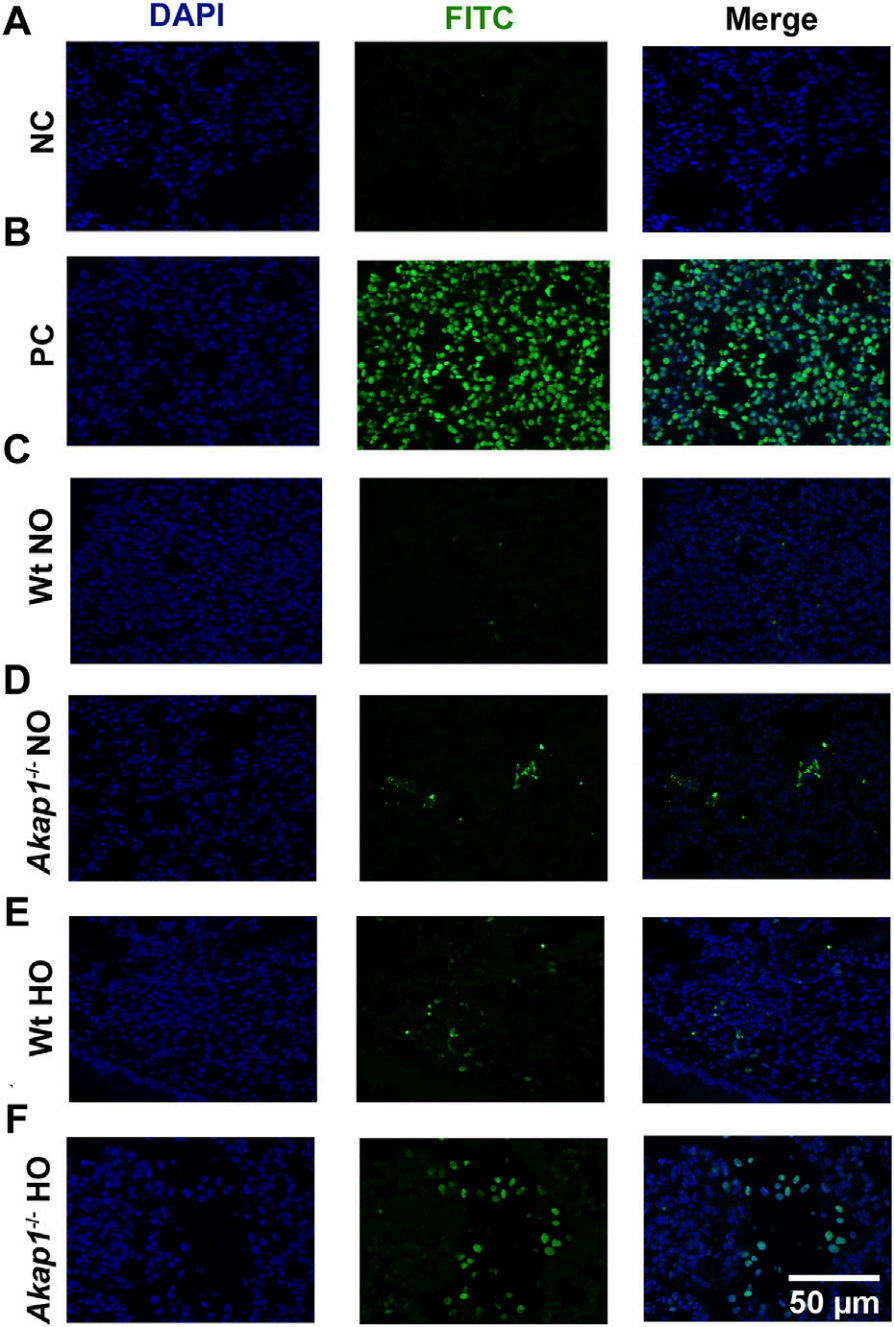
Increase in TUNEL positive cells in *Akap1* ^−/−^ mice exposed to hyperoxia. **(A)** Representative images of negative control (NC) and **(B)** positive control (PC), respectively. **(C,D)** Representative images of Wt normoxia and *Akap1* ^−/−^ normoxia lung sections stained with FITC and DAPI. **(E,F)** Representative images of Wt hyperoxia and *Akap1* ^−/−^ hyperoxia lung sections stained with FITC and DAPI. Nuclei stained with DAPI (blue) and TUNEL positive cells stained with FITC (green). Magnification: ×400, scale bar: 50 μm. NO = normoxia and HO = hyperoxia.

To evaluate if apoptosis is increasing in *Akap1* ^−/−^ mice exposed to hyperoxia versus Wt hyperoxia control, TUNEL staining was performed. The negative control showed no TUNEL positive cells (Figure 7A), whereas TUNEL positive cells were observed in the positive control (Figure 7B). In the lung section from the Wt mice exposed to normoxia, no TUNEL staining was observed (Figure 7C). Interestingly, TUNEL positive cells were detected in lung sections in *Akap1* ^−/−^ exposed to normoxia (Figure 7D). Lung section from Wt mice exposed to 48 h of hyperoxia also showed an increase in TUNEL positive cells versus Wt normoxia control (Figure 7E). TUNEL positive cells increased in lung section from *Akap1* ^−/−^ lung mice exposed to hyperoxia relative to Wt hyperoxia group (Figure 7F).

## Discussion

The key finding of this study is that *Akap1* deletion exacerbates HALI *via* impaired mitochondrial dysfunction. At the transcriptome level, hyperoxia induced an increase in transcripts encoding proteins of ETC and TCA cycle in *Akap1* ^*−/−*^ mice. Further, *Akap1* ^*−/−*^ mice subjected to oxidant injury exhibit impaired aconitase, fumarase and citrate activities. The mechanism of mitochondrial dysfunction attributed to AKAP1 loss involves Drp1 dephosphorylation at Ser637 followed by Akt inactivation and Bax activation. In addition, there is an increase in the expression of antioxidant HO-1 in the alveolar macrophages of *Akap1* ^*−/−*^ mice.

Previous research from our laboratory demonstrates that AKAP1 loss exacerbates HALI. Hyperoxia exposure in *Akap1* ^*−/−*^ mice increases mitophagy and causes mitochondrial aberrations (Narala et al., 2018). A 2021 transcriptome study reports that oxidant injury impairs neonatal lung development via activation of inflammatory pathways (Hurskainen et al., 2021). Therefore, to understand the mechanism of hyperoxia-induced mitochondrial dysfunction in adult *Akap1* ^*−/−*^ mice lungs, we performed a transcriptome analysis. Loss of AKAP1 was found to be a main driver in hyperoxia-induced transcriptional changes in ETC and TCA cycle implicating AKAP1 in mitochondrial bioenergetics. The increase in transcripts encoding Complex I to V proteins in *Akap1* ^*−/−*^ mice exposed to hyperoxia suggests the possibility of an endogenous defense mechanism to counteract oxidative stress.

In this study, OXPHOS Complex I expression decreased significantly in *Akap1* ^*−/−*^ and *Wt* mice in response to oxidative stress, whereas OXPHOS Complexes II, III, IV or V expression were unaffected. In contrast, long term cigarette smoke exposure (CSE) in human bronchial epithelial cell line BEAS-2B significantly shows an increase in the expression of OXPHOS Complexes II, III and V (Hoffmann et al., 2013). Other independent studies showed that CSE exposure in primay lung cells including SAEC and NHBE altered the expression of OXPHOS Complexes I-IV, whereas only complexes I and II were affected in BEAS2B cells (Sundar et al., 2019). Our present study correlates with this study as Complex I expression was affected under oxidative stress. The Complex I activity is affected only in Wt mice and not in *Akap1* ^−/−^ mice exposed to hyperoxia. In another study, hyperoxia exposure in bronchopulmonary dysplasia (BPD) mice model induced a decrease in OXPHOS Complex I and II activities in neonatal lungs (Das, 2013). Similarly, a decrease in ETC activity in cystic fibrosis is connected to Complex I dysfunction and associates with an increase in mitochondrial calcium and oxygen consumption rates (Feigal and Shapiro, 1979; Shapiro et al., 1979; Valdivieso et al., 2007). In addition, lung epithelial cells exposed to high doses of cigarette smoke showed reduced OXPHOS Complex I and II activities (van der Toorn et al., 2007). This suggests that Akap1 does not impact Complex I activity in response to oxidative stress.

Type II AECs have the highest number and size of mitochondria relative to other lung cells (Massaro et al., 1975). Further, OXPHOS metabolism, a primary energy source in Type II AECs, contributes to oxidant-induced mitochondrial dysfunction susceptibility (Schumacker et al., 2014). In this study, we found that the transcripts encoding TCA cycle proteins, namely citrate synthase, aconitase 2 and succinate dehydrogenase, significantly increased in *Akap1* ^*−/−*^ mice and were unaffected in *Wt* mice in response to oxidant injury. This indicates an endogenous compensatory mechanism in *Akap1* ^*−/−*^ mice to counteract the loss of ATP and mitochondrial respiration. Interestingly, aconitase activity significantly decreased in *Akap1* ^*−/−*^ mice exposed to hyperoxia versus the *Wt* hyperoxia group suggesting that AKAP1 loss deregulates this energy producing pathway (TCA) during oxidative stress. Hyperoxia causes oxidative modification of aconitase (carbonylation) in flies and aconitase activity decreases significantly only after 72 h of exposure (Yan et al., 1997). An increase in aconitase activity in *Wt* mice exposed to 48 h of hyperoxia suggests that at this time point, the presence of AKAP1 drives this response. It is plausible that after 72 h of hyperoxia exposure, *Wt* mice may show decreased aconitase activity. Even in the neuronal cells, AKAP1 loss causes OXPHOS Complex II dysfunction (Flippo et al., 2018). Similarly, loss of AKAP1 in RGCs leads to an increase in OXPHOS Complex II and a decrease in Complex III-V activities (Edwards et al., 2020). These observations implicate a broad role for AKAP1 in regulating OXPHOS function in lungs as well as brain.

Type II AECs initiate mitochondrial biogenesis in response to oxidative stress to maintain mitochondrial integrity (Schumacker et al., 2014). In our study, perturbation of mitochondrial dynamics occurred in cells lacking AKAP1 and subjected to oxidative stress. In *Wt* mice subjected to oxidative insult, there was an increase in Drp1 phosphorylation at Ser637. This may be a response to initiate mitochondrial fusion. In contrast, in *Akap1* ^*−/−*^ mice exposed to hyperoxia, a significant reduction in Drp1 phosphorylation at Ser637 in lungs suggests a failure to induce mitochondrial fusion. A 2018 study shows that the loss of AKAP1 promoted mitochondrial fission due to reduced Drp1 phosphorylation at Ser637, localized Drp1 to the brain mitochondria and exacerbated stroke in a murine model of transient cerebral ischemia (Flippo et al., 2018). Likewise, *Akap1* deletion in mice and glaucomatous RGCs reduced phosphorylation of Drp1 at Ser637 and triggered mitochondrial fission (Edwards et al., 2020). These studies indicate that Drp1 phosphorylation at Ser637 by PKA is a critical step for mitochondrial fusion, whereas dephosphorylation at Ser637 induces mitochondrial fission. It also indicates that overexpression of AKAP1 may increase cell survival (Liu et al., 2020). In a 2021 study, mitoquinone (MitoQ) exerted a protective effect against LPS-induced ALI via regulating the Drp1-mediated mitochondrial fission and activating the Nrf2 pathway (Hou et al., 2021).

Hyperoxia-induced oxidative stress activates the Nrf2 pathway (Reddy et al., 2009) and is one of the defense mechanisms against oxidative stress, thus promoting cell survival (Loboda et al., 2016; Bellezza et al., 2018). HO-1, a downstream target of Nrf2, breaks down haem and protects against oxidative stress. The expression of Nrf2 target HO-1 significantly increased in response to AKAP1 loss and hyperoxia versus hyperoxia alone suggesting an additive effect of *Akap1* deletion and oxidative stress. The CD68 ^+^ alveolar macrophages predominantly express HO-1 in the lung sections of *Akap1* ^*−/−*^ mice exposed to hyperoxia. HO-1 induction in *Akap1* ^*−/−*^ mice post-hyperoxia suggests a robust endogenous response. It implies that Akap1 regulates the Nrf2 pathway. Similarly, HO-1 expression is increased in the alveolar macrophages of cigarette smokers suggesting an oxidative stress response (Maestrelli et al., 2001).

Activation of the Akt/PI3K pathway promotes cell survival (Chua et al., 2009). Adenovirus mediated expression of constitutively active form of Akt in mice exposed to hyperoxia delayed the onset of ALI and prolonged cell survival (Liu et al., 2020). Further, AKAP1 deficiency in cardiomyocytes is associated with Akt inactivation resulting in increased apoptosis and dysfunction (Schiattarella et al., 2018). In another study, the loss of AKAP1 decreases Akt phosphorylation at Ser473 and activates the apoptotic pathway in the retina (Edwards et al., 2020). In this study global deletion of *Akap1* significantly decreases Akt activation in murine lungs. This suggests activation of the apoptotic pathway due to Akt inactivation (Kennedy et al., 1999). Hyperoxia-activated protein kinases cause Bax activation, translocation to mitochondria and apoptosis (Kolliputi and Waxman, 2009). IL-6 overexpression protects against oxidant injury via PI3K/Akt-mediated phosphorylation at Ser184 and inhibits Bax translocation and dimerization to the mitochondrial membrane (Kolliputi and Waxman, 2009). This study suggests that AKAP1 loss induces Akt inactivation via dephosphorylation at Ser473 and causes an increase in Bax expression even under normoxia. In contrast, AKAP1 loss and oxidant injury causes an increase in Akt phosphorylation at Ser473. Akt activation depends on phosphorylation at Thr308 and Ser473 residues and total Akt levels (Alessi et al., 1996). In this study, Akt phosphorylation was analyzed only at Ser473. Further, Akap1 deletion and oxidant injury exacerbates ER stress in mice (Sidramagowda Patil et al., 2022). This suggests that the ER stress in *Akap1* ^*−/−*^ mice exposed to hyperoxia may induce phosphorylation of Ser473 via GRP78 and, in turn, defines Akt target specificities (Yung et al., 2011). Preliminary data generated in our laboratory revealed that the lungs of the ARDS patients had reduced levels of AKAP1 protein (unpublished observations) suggesting that this may impact mitochondrial function. In light of our current findings, AKAP1 overexpression and modulation of Drp1 phosphorylation at Ser637 is an important therapeutic strategy for acute lung injury.

## Data Availability

The datasets presented in this study can be found in online repositories. The names of the repository/repositories and accession numbers can be found below: Gene expression Omnibus (GEO); GSE173295.

## Abbreviations

AKAP1, A-kinase anchoring protein 1; HALI, Hyperoxia induced acute lung injury; ALI, Acute lung injury; DEG, Differentially expressed genes; ETC, Electron transport chain; TCA, Tricarboxylic acid cycle; IPA, Ingenuity pathway analysis; OXPHOS, Oxidative phosphorylation; Drp1, Dynamin-related protein 1; SOD2, Superoxide dismutase 2; HO-1, Haem oxygenase 1; ROS, Reactive oxygen species.
