# Supplementary material for: A-Kinase Anchor Protein 1 deficiency causes mitochondrial dysfunction in mouse model of hyperoxia induced acute lung injury
Source: Front Pharmacol. 2022 Oct 3;13:980723. doi: 10.3389/fphar.2022.980723 (PMC9574061; doi:10.3389/fphar.2022.980723)
Supplement: Supplementary file 3 [file Presentation1.pdf]

Supplemental data

Supplementary Table 1. Differentially expressed genes (DEGs) in *Akap1*$^{-/-}$ hyperoxia versus *Akap1*$^{-/-}$ normoxia controls.

Supplementary Table 2. Differentially expressed genes (DEGs) in Wt hyperoxia versus Wt normoxia controls.

Supplementary Table 3. Differentially expressed genes in the OXPHOS pathway from *Wt* and *Akap1*$^{-/-}$ (normoxia vs. hyperoxia) datasets.

Supplementary Table 4: Differentially expressed genes (DEGs) in Wt normoxia versus *Akap1*$^{-/-}$ normoxia group.

Supplementary Table 5: Differentially expressed genes (DEGs) in Wt hyperoxia versus *Akap1*$^{-/-}$ hyperoxia group.

Supplementary Figure 1. Phenotypic characterization of *Akap1*$^{-/-}$ mice lungs. (A) Genotyping shows PCR products separated on 1.0% Agarose gel and stained with ethidium bromide. Wt band: 600 bp and KO band: 350 bp. Genotyping data of mice groups used for RNA-seq experiments. (B) Akap1 gene expression in Wt normoxia (NO), *Akap1*$^{-/-}$ normoxia (NO), Wt hyperoxia (HO) and *Akap1*$^{-/-}$ hyperoxia (HO) groups from RNA-seq data. Cpm is counts per million reads. Data represented as mean ± S.E.M. n=3 mice per group. One-way ANOVA and post-hoc Tukey test. * $p<0.05$ versus *Akap1*$^{-/-}$ NO control. (C) Western blot analysis of AKAP1 expression in lung lysates from *Wt* and *Akap1*$^{-/-}$ mice exposed to normoxia and hyperoxia. β-Actin is used as a loading control. Quantitation of Akap1 expression after normalization to β-Actin. Data represented as mean ± S.E.M. n=3 mice per group. One-way ANOVA and post-hoc Tukey test. *** $p<0.0001$ versus Wt normoxia and *** $p<0.0001$ versus Wt hyperoxia. (D) Hematoxylin and Eosin stained images of Wt and *Akap1*$^{-/-}$ Normoxia and hyperoxia lungs. Br: Bronchiole, Alv: Alveolar region. Arrowhead shows immune cell infiltration. Magnification: 40x . Scale bar: 100 µM.

Supplementary Figure 2. Integrated Genome Viewer (IGV) of *Akap1* gene. Reads shown for Exons 1-11 right to left. Exon 2 is boxed. Reads shown for Wt (n=6) and *Akap1*$^{-/-}$ (n=6) mice samples.
