# Supplementary figures and images for: A-Kinase Anchor Protein 1 deficiency causes mitochondrial dysfunction in mouse model of hyperoxia induced acute lung injury
Source: Front Pharmacol. 2022 Oct 3;13:980723. doi: 10.3389/fphar.2022.980723 (PMC9574061; doi:10.3389/fphar.2022.980723)

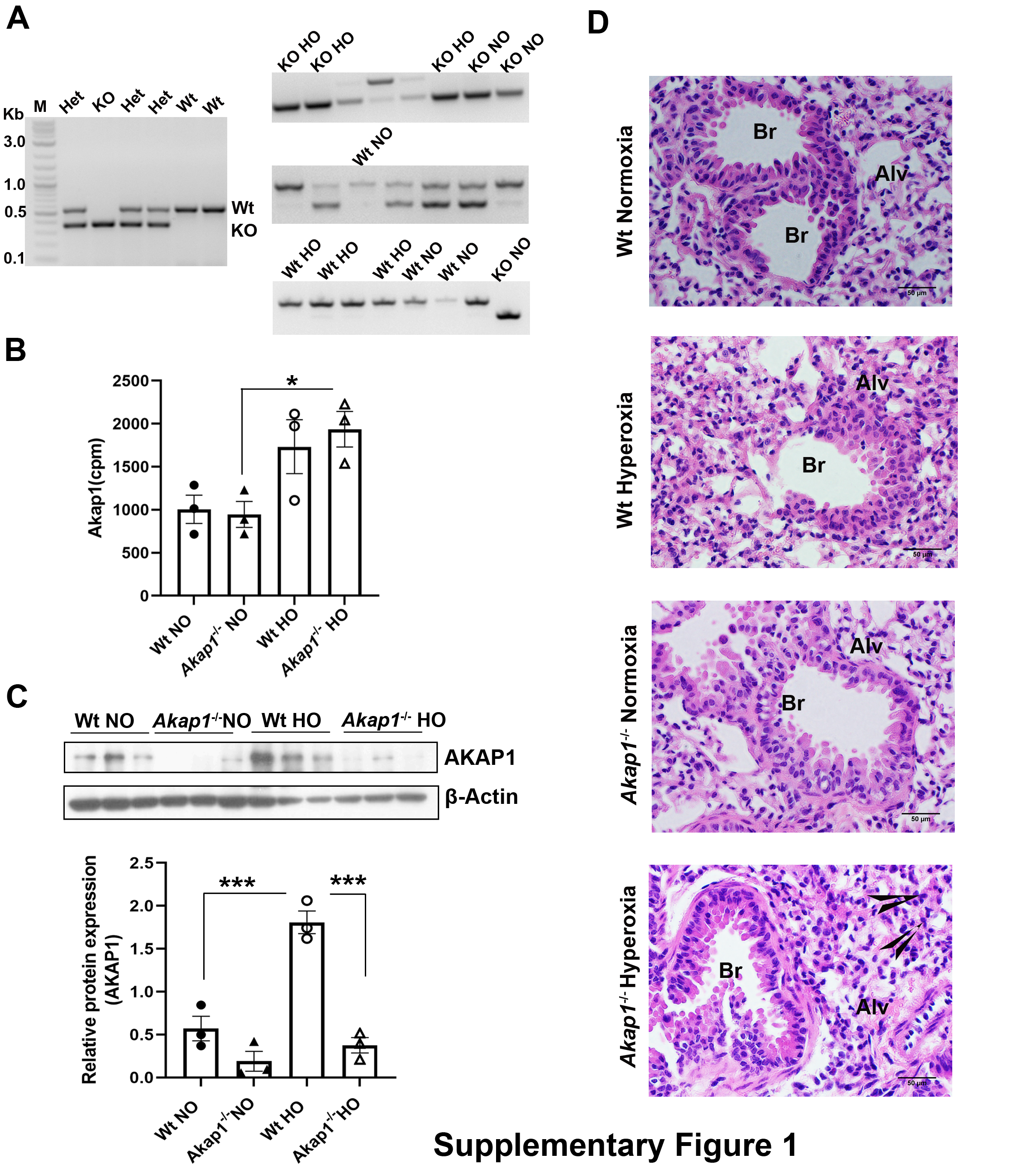

Supplement: Supplementary file 4 [file Image1.TIF]

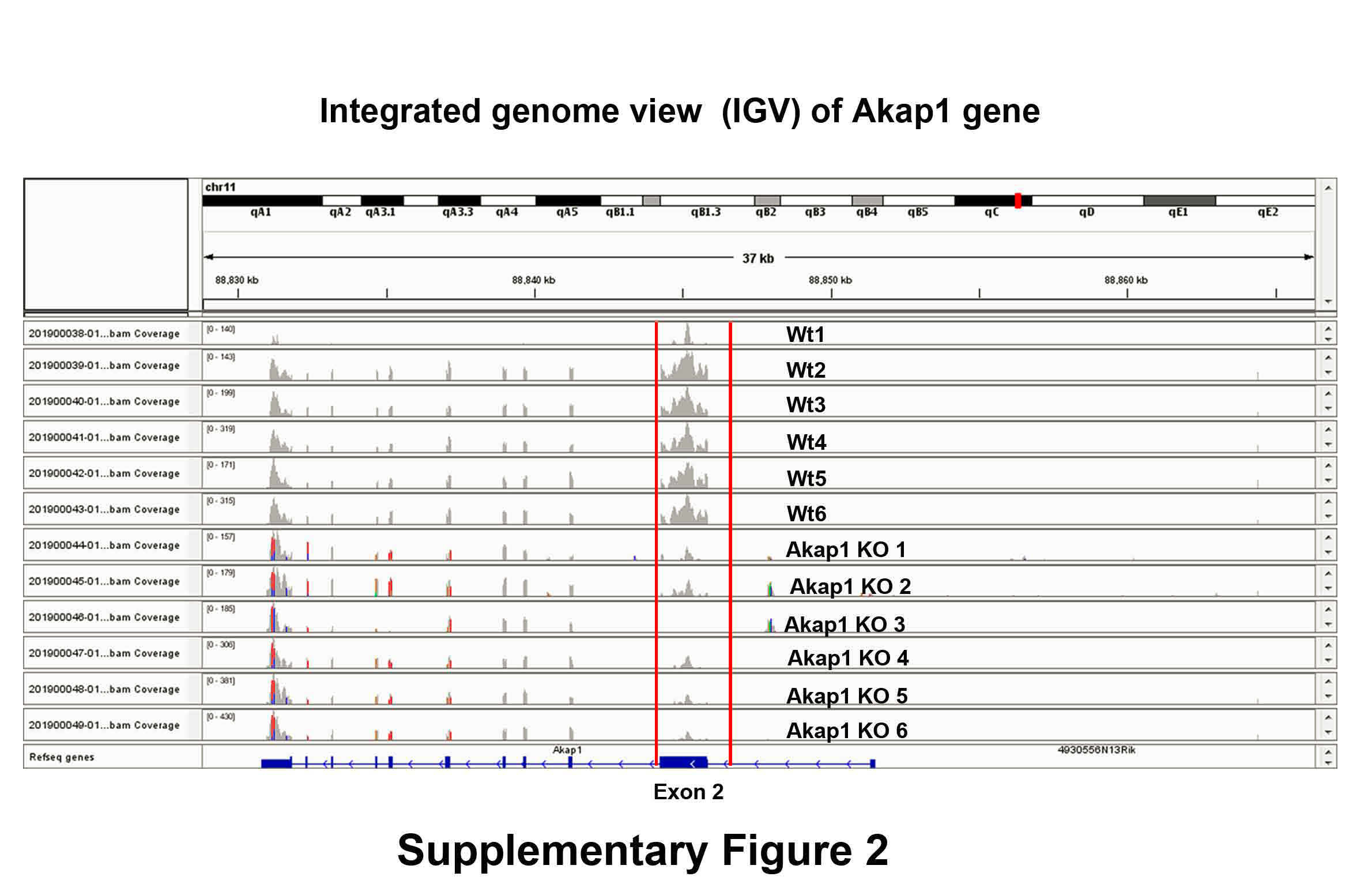

Supplement: Supplementary file 8 [file Image2.TIFF]
